# A Case of Urothelial Carcinoma With Squamous Differentiation of the Bladder, Infiltrating the Small Intestine and Rectus Abdominis, Treated With the Use of a Short Gracilis Myocutaneous Flap

**DOI:** 10.1002/iju5.70077

**Published:** 2025-07-21

**Authors:** Kazuto Imai, Norihiko Masuda, Tatsuya Hazama, Kanji Nagahama, Takashi Ito, Takakazu Matsushita, Yoko Muneta, Tadashi Inoue, Toshiya Akao

**Affiliations:** ^1^ The Department of Urology Rakuwakai Otowa Hospital Kyoto Japan; ^2^ The Department of Surgery Rakuwakai Otowa Hospital Kyoto Japan; ^3^ The Department of Plastic Surgery Rakuwakai Otowa Hospital Kyoto Japan

**Keywords:** local invasion, myocutaneous flap, squamous differentiation, urothelial carcinoma

## Abstract

**Introduction:**

Squamous differentiation (SD) occurs in up to 20% of muscle invasive bladder cancers.

**Case Presentation:**

An 85‐year‐old man with an intrapelvic mass invading the bladder, small intestine, and rectus abdominis presented to our department. Cystoscopy showed a necrotic mass at the dome of the bladder. Histopathological examination of specimens from transurethral resection indicated squamous cell carcinoma. Radical cystectomy with resection of the small intestine and rectus abdominis, and reconstruction of the abdominal wall using a left short gracilis myocutaneous flap was performed. The histopathology showed squamous cell carcinoma and urothelial carcinoma (UC) components; findings consisted of UC with SD. The patient received appropriate flap management and treatment for postoperative complications and was discharged on postoperative Day 100.

**Conclusion:**

To our knowledge, this is the first report of an invasive bladder cancer with SD treated with the use of a short gracilis myocutaneous flap.

**Summary**

- We report a case of urothelial carcinoma with squamous differentiation of the bladder invading the small intestine and rectus abdominis, and it was treated with the use of a short gracilis myocutaneous flap.

**Abbreviations**
CT: computed tomography
MIBC: muscle invasive bladder cancer
PICC: peripherally inserted central catheter
RC: radical cystectomy
SCC: squamous cell carcinoma
SD: squamous differentiation
TUR‐BT: transurethral resection of bladder tumor
UC: urothelial carcinoma
UCSD: urothelial carcinoma with squamous differentiation

## 1 Introduction

Squamous differentiation (SD) occurs in up to 20% of muscle invasive bladder cancer (MIBC) cases and is associated with advanced tumor stage [1]. Because its presence in urothelial carcinoma (UC) was an independent predictor of local recurrence after radical cystectomy (RC) [2], extensive resection should be considered to achieve negative margins in selected cases. We report a case of urothelial carcinoma with squamous differentiation (UCSD) of the bladder invading the small intestine and rectus abdominis and was treated with the use of a short gracilis myocutaneous flap.

## 2 Case Presentation

**FIGURE 1:**
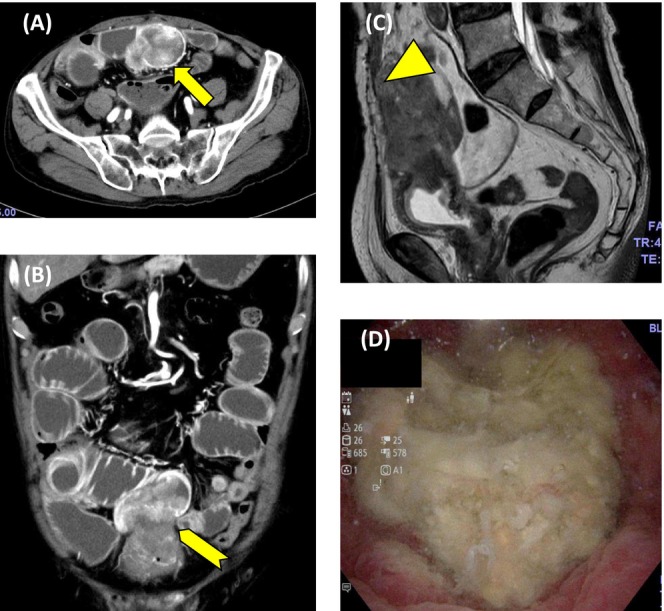
Contrast‐enhanced CT revealed a peripherally enhancing mass (A, arrow), which involved the dome of the bladder and the ileum (B, arrowhead). Magnetic resonance imaging suggested that the mass invaded the rectus abdominis (C, triangle). Cystoscopy showed a necrotic mass at the dome of the bladder (D).

**FIGURE 2:**
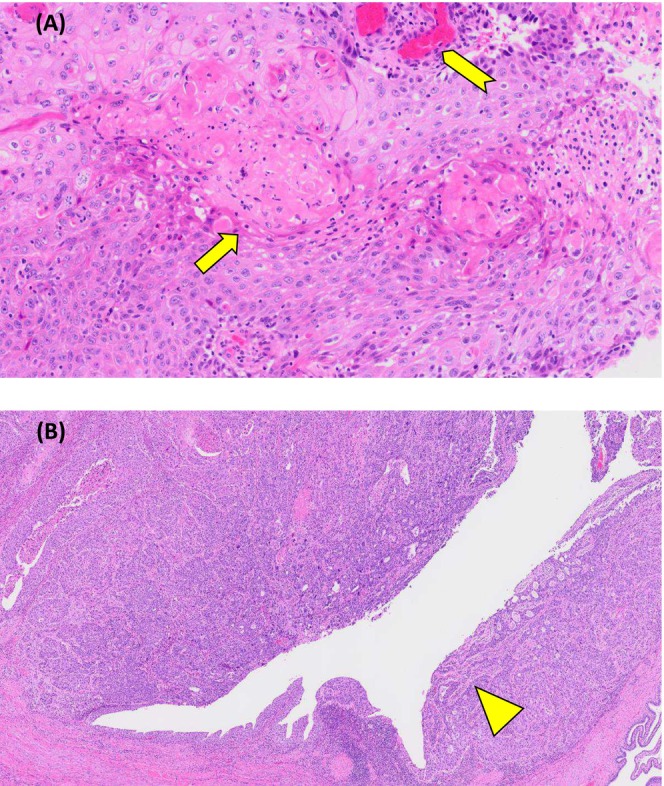
Histopathological examination of the tumor specimens showed solid and papillary proliferation formation of keratin pearls (arrow) and focal necrosis (arrowhead) (A). The histopathological examination of the removed specimen showed extensive areas of keratinization and necrosis, along with urothelial carcinoma components (triangle) (B).

**FIGURE 3:**
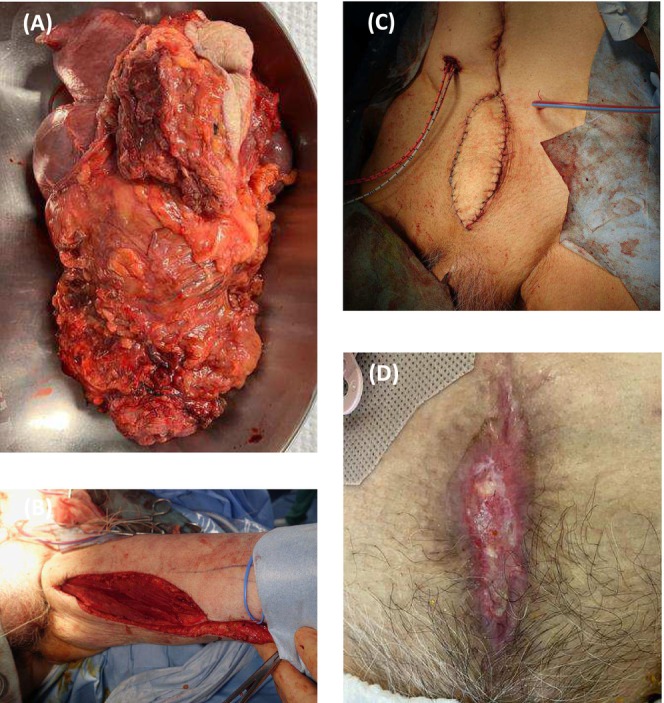
Photograph of the mass, which was removed *en bloc*, including the bladder, portion of the small intestine, rectus abdominis, and umbilicus (A). The left short gracilis myocutaneous flap dissection was carried out in standard fashion and brought into the resultant infra‐umbilical abdominal wall defect through a tunnel created subcutaneously through the left inguinal region (B, C). By the time of discharge, the flap had successfully engrafted with accompanying scar formation (D).

An 85‐year‐old man presented to the emergency department with abdominal pain. Computed tomography (CT) of the abdomen showed an intrapelvic mass causing obstruction of the small intestine. Contrast‐enhanced CT revealed a peripherally enhancing mass involving the dome of the bladder and the ileum (Figure 1A,B); its origin could not be determined. No lymph node or distant metastases were identified. Magnetic resonance imaging suggested that the mass invaded the rectus abdominis (Figure 1C). Cystoscopy showed a necrotic mass at the dome of the bladder (Figure 1D). An abdominal CT performed 9 years ago during an evaluation for lower gastrointestinal bleeding showed no evidence of urachal remnant. Due to obstruction of the small intestine, the patient was admitted on an emergency basis, and transurethral resection of bladder tumor (TUR‐BT) was performed on hospital Day 10. Histopathological examination of the tumor specimens showed solid and papillary proliferation with formation of keratin pearls and focal necrosis. It supported a diagnosis of squamous cell carcinoma (SCC), and no UC component was identified (Figure 2A). The serum SCC level after TUR‐BT was 9.4 ng/mL. Because SCC of the small intestine is quite rare, the primary disease was considered more likely to be SCC or UCSD of the bladder. As the removal of the rectus abdominis would result in a large soft tissue defect of the lower abdominal wall, a rotational myocutaneous flap was selected for the reconstruction. Open RC with resection of small intestine and rectus abdominis, bilateral cutaneous ureterostomy, and reconstruction of the abdominal wall using a left short gracilis myocutaneous flap were performed on hospital Day 27. The mass was removed *en bloc*, including the umbilicus (Figure 3A). The left short gracilis myocutaneous flap dissection was carried out in standard fashion and brought into the resultant infra‐umbilical abdominal wall defect through a tunnel created subcutaneously through the left inguinal region (Figure 3B,C). Histopathologically, the resected tumor showed extensive areas of keratinization and necrosis, along with UC component, which indicated that UCSD of the bladder was the primary disease (Figure 2B). No cystic components or glandular structures suggestive of urachal carcinoma were observed. The resection margins were negative. After the surgery, the patient received continuous infusions of low molecular dextran and prostaglandin E1 for 7 days. Subsequently, topical prostaglandin E1 ointment was applied to the flap area, and povidone‐iodine sugar paste ointment was applied to the flap harvest site. Pus discharge associated with skin necrosis was observed and debridement was initiated. On postoperative Day 12, hyperbaric oxygen therapy was administered for 10 days as part of the treatment of paralytic ileus. By the time of discharge, the flap had successfully engrafted with accompanying scar formation (Figure 3D). The flap harvest site had completely healed.

Because of the postoperative paralytic ileus, the oral intake was restricted and a gastric tube, ileus tube, and peripherally inserted central catheter (PICC) were placed subsequently. Following PICC placement, the patient developed suppurative thrombophlebitis on postoperative Day 25, which required antibiotic and anticoagulant therapy. Candidemia secondary to ureteral stent obstruction also developed, which was treated with antifungal agents. The patient's condition gradually improved with the slow reintroduction of oral intake and prolonged administration of antibiotics, anticoagulants, and antifungal agents, allowing for discharge on postoperative Day 100. The serum SCC level after RC was 2.3 ng/mL, and no recurrent lesions were observed on CT on postoperative Day 141.

## 3 Discussion

SD is the most common variant, occurring in up to 20% of MIBC cases [1]. In our case, no UC component was identified in the TUR‐BT specimens, which caused the indistinctness of the primary origin. Small intestine cancers comprise only 1%–2% of all gastrointestinal malignancies [3], which means that SCC of the small intestine is a more rare entity. Although the small intestine, especially the ileum, is the most common site for gastrointestinal tract metastasis from lung cancer including SCC [4], no lung tumor was identified in our case. SCC of the bladder is also rare, accounting for < 5% of all bladder cancers [5]. While risk factors for SCC of the bladder vary between geographic regions, they include chronic or recurrent urinary tract infection, catheter use, smoking history, and others [6]. We did not identify any such risk factors in our case. Although SCC and UCSD have similar oncological outcomes, they are distinct pathological entities [7]. Given the epidemiological background of our case, UCSD of the bladder was considered the most likely preoperative diagnosis. While open RC with resection of small intestine and rectus abdominis was performed to achieve negative margins, we probably should have performed lymph node dissection as well, as the rate of nodal metastasis is significantly higher with UCSD with pure UC [8]. However, it was omitted due to the patient's decline in performance status resulting from prolonged fasting associated with small intestine ileus, the high invasiveness of surgical procedures, and the absence of preoperative lymphadenopathy.

Abdominal wall deficiency can be classified into two groups, the hernia, an abdominal wall weakness limited to the fascia, and the full‐thickness abdominal wall defect which requires soft‐tissue coverage. Complete stability can be achieved in hernia repair by using synthetic meshes or autologous grafts [9, 10, 11, 12]. The goals of reconstruction of the full‐thickness abdominal wall include reestablishment of the functional integrity with adequate soft tissue coverage [13]. The main options for repairing lower abdominal wall defects include pedicled muscle or musculocutaneous flaps harvested from the tensor fasciae lata, rectus femoris, or sartorius [14, 15, 16]. In our case, a short gracilis myocutaneous flap was used based on the size of the defect and our institutional experience. The reconstruction was successful, and muscle strength in the left lower extremity was preserved. To our knowledge, this is the first report of a UCSD of the bladder treated with the use of a short gracilis myocutaneous flap. A curative resection was achieved through a multidisciplinary surgical approach involving urology, general surgery, and plastic surgery.

## Ethics Statement

The authors have nothing to report.

## Consent

The authors have nothing to report.

## Conflicts of Interest

The authors declare no conflicts of interest.
